# The Use of Motion Analysis as Particle Biomarkers in Lensless Optofluidic Projection Imaging for Point of Care Urine Analysis

**DOI:** 10.1038/s41598-019-53477-8

**Published:** 2019-11-21

**Authors:** Jessica Kun, Marek Smieja, Bo Xiong, Leyla Soleymani, Qiyin Fang

**Affiliations:** 1 School of Biomedical Engineering, McMaster University, Hamilton, ON Canada; 2 Department of Pathology and Laboratory Medicine, McMaster University, Hamilton, ON Canada; 3 Department of Engineering Physics, McMaster University, Hamilton, ON Canada

**Keywords:** Imaging and sensing, Diagnostic markers

## Abstract

Urine testing is an essential clinical diagnostic tool. The presence of urine sediments, typically analyzed through microscopic urinalysis or cell culture, can be indicative of many diseases, including bacterial, parasitic, and yeast infections, as well as more serious conditions like bladder cancer. Current urine analysis diagnostic methods are usually centralized and limited by high cost, inconvenience, and poor sensitivity. Here, we developed a lensless projection imaging optofluidic platform with motion-based particle analysis to rapidly detect urinary constituents without the need for concentration or amplification through culture. A removable microfluidics channel ensures that urine samples do not cross contaminate and the lens-free projection video is captured and processed by a low-cost integrated microcomputer. A motion tracking and analysis algorithm is developed to identify and track moving objects in the flow. Their motion characteristics are used as biomarkers to detect different urine species in near real-time. The results show that this technology is capable of detection of red and white blood cells, *Trichomonas vaginalis*, crystals, casts, yeast and bacteria. This cost-effective device has the potential to be implemented for timely, point-of-care detection of a wide range of disorders in hospitals, clinics, long-term care homes, and in resource-limited regions.

## Introduction

Urinalysis is a valuable tool for the diagnosis of various conditions through physical, chemical, and microscopic analysis. Physical analysis is the observation of urine’s physical characteristics, whereas chemical and microscopic analysis tests for the presence of chemical analytes^1–3^ and urine sediments (0.5–500 μm) respectively^4^. Simerville *et al*. provides a comprehensive list of analytes, sediments, and the current clinical methods of analysis^4^.

Generally, in microscopic urinalysis, targeted sediments (listed in Table 1) can be identified through morphological features by a technician after centrifuging the urine to obtain a concentrated sample. In the case of microorganisms, a stain can be used for identification through microscopy, but the gold standard is tissue culture^4^. However, outpatient clinics and even clinical laboratory collection sites do not normally have these specialized instruments or trained technicians to perform these tests. As a result, samples are sent off to a centralized facility for processing, e.g. at the Hamilton Regional Lab Medicine Program, which can have over a thousand samples to process per week. Such processing is efficient for large number of samples, but some issues exist. For example, it is particularly detrimental in the case of trichomoniasis, an infection caused by a parasite known as *Trichomonas vaginalis* (TV). Trichomoniasis is estimated to be the most common non-viral sexually transmitted infection (STI) with 276.4 million cases worldwide^5^. It is often underdiagnosed due to the lack of a conventional test^6^ despite being associated with poor birth outcomes^7^ such as low birth weight, preterm delivery, and intellectual disability in children^5,8^. The current gold standard for trichomoniasis diagnosis is culture followed by wet mount microscopy, a procedure not easily done on-site. However, TV is only viable for approximately four hours after leaving the body so by the time the samples reach a centralized lab, they may have died. This makes diagnosis more difficult as one of the defining characteristics of *Trichomonas vaginalis* is their unique motility^6^. Point-of-care tests have also been developed for the diagnosis of trichomoniasis, however it remains too costly to implement^9^.

**Table 1:** A list of the different components of urine and their sizes, the subsequent diagnosis if found. Information was derived from Simerville *et al*.^4^.

Component	Diagnosis	Size (um)
Leukocytes (White Blood Cells)	- Normally, men have <2 WBC/HPF (high powered field) and women <5WBC/HPF	10–12
Erythrocytes (Red Blood Cells)	->3 RBC/HPF in two of three urine samples suggests hematuria - If RBCs are dysmorphic, patient may have glomerular disease.	6–8
Epithelial cells	- Squamous epithelial cells suggests contamination - Transitional epithelial cells is normal - Renal tubule cells indicates significant renal pathology	15+
Casts	- Casts are used to localize disease to a specific location in the genitourinary tract depending on their composition - Hyaline casts can be associated with pyelonephritis or chronic renal disease. A full list of casts and associated conditions can be found in Simerville *et al*.^4^.	15+
Crystals	- Calcium oxalate crystals are normal - Uric acid crystals are normal - Triple phosphate crystals are associated with UTIs caused by Proteus - Cystine crystals are associated with cystinuria	15+
Bacteriuria	- In asymptomatic females 5 bacteria/HPF (roughly 100,000 colony forming units (CFU) per mL) represents asymptomatic bacteriuria - In symptomatic patients, 100 CFU per mL suggests UTI - In males, presence of bacteria is abnormal and culture should be obtained	1–2
Parasites	- Although less common, parasitic infections can also be detected in the urine. The two most common parasites that give rise to urological disorders are schistosomiasis (1 mm length) and Trichomonas vaginalis (10 μm length). *Trichomonas vaginalis* often give rise to renal and lower urinary tract diseases, and Schistosomiasis leads to permanent urogenital problems and renal failure^39^.	10–1000
Yeast	- The presence of budding yeast, Candida albicans, can be an indication of a yeast infection. They can be single, budding, or branched based on the severity of the infection.	5–10
Clue cells	- Bacteria coats epithelial cells in an infection known as bacterial vaginosis. These cells are known as clue cells under the microscope and are a good indication of infection. Severity varies based on the extent of bacterial coverage.	15+

Another example of a potential condition is a urinary tract infection (UTI), an infection caused by the presence of bacteria in the urinary tract with prevalence among communities and hospitals. UTIs affect almost 50% of the population at least once in their lifetime, leading to an annual health care cost of approximately $3.5 billion in the US^10^, $1.6 billion of which contributes to the administration of antibiotics^11^, enhancing the risk of antibiotic resistance^12^. It takes 48 hours for urine to be cultured and 70% of samples come back negative^13^. A third particulate that can be found in urine is red blood cells (RBCs). Blood in the urine is known as hematuria and can be a symptom of a large range of conditions, including kidney disease, cancer, etc^4^. The clinical definition of hematuria is >3 RBCs per high power field meaning each sample must be tested under a microscope by a trained technician, a time consuming and inconvenient process.

To improve the efficacy of urinalysis, flow cytometry techniques been applied^11^ as a preliminary screening tool that aims to reduce the number of samples cultured, reducing the workload, time, and costs in large laboratories^14^. The use of flow cytometry as a pre-screening tool has presented a 28–60% reduction in the number of cultured samples^11^. In addition to saving cost and resources, by immediately receiving a negative result, physicians avoid prescribing unnecessary antibiotics and can go on to providing a more accurate diagnosis quicker. Savings of $239–$306 USD per 100 samples have also been reported, indicating the use of a flow cytometer is also cost efficient^15^. Nevertheless, flow cytometry has its limitations as the samples must be labelled, and in image-based flow cytometry the specimens are at risk of being imaged out of focus due to a short depth of field^16^. Flow cytometers have a large benchtop footprint and are expensive. Thus, they are typically implemented at the level of the centralized processing facility, which often sees a delay between sample collection and processing due to the transportation of the samples. For the most accurate results, the urine must be examined within two hours as longer delay times often cause unreliable results^4^. A platform that can be integrated into the physician’s office would ensure that the sample is processed in real time. The workflow for urinalysis can benefit from a less expensive and more time-efficient diagnostic tool.

Lensless, or lens-free, imaging devices offer a different approach to detecting small particles in large fluid volumes. Lensless microscopy records the image of the sample on the detector without any intervening lenses. Imaging without lenses offers advantages over cell culture and traditional microscopy, including low-cost, large field of view, and portability, which inherently leads to high throughput while maintaining sub-micron resolution. It is particularly well suited to analysis applications in which a large area or volume must be screened in order to determine whether a sample is positive or negative, making it ideal for urine analysis. Lensless imaging can be used in combination with microfluidics to make a cost-effective and portable device that can evaluate milliliters of liquid for microscopic specimen, in under an hour, without the need for centrifugation. Shadow imaging and holographic imaging are two lensless techniques resulting in a bright field image^17^. The resolution of the images attained from these modalities is limited to twice the size of the pixel and depends on the sample-sensor distance^17^. An advantage of shadow imaging is that the images acquired do not require post processing or reconstruction. It is normally well suited for the imaging of biological specimen, in which the samples have some degree of transparency. A number of lensless imaging devices have been fabricated as an alternative to standardized health care screens. These include blood counting and analysis^18^, pap smear analysis^19^, sperm motility analysis^20^, and so on. Recently, Zhang *et al*. developed a lensless holographic imaging device for the analysis of parasites in cerebrospinal fluid^21^. Their device is adaptable to Trichomonas vaginalis detection, though further testing must be done in spiked urine samples. The device uses holographic phase imaging to scan and analyze 3 ml of fluid in 20 minutes. The limit of detection was found to be 10 parasites per milliliter of whole blood. It is evident that lensless imaging has a lot of potential for health care monitoring. This paper aims to demonstrate that shadow imaging is well-suited to the application of urine analysis, especially in combination with motion analysis of urine sediments.

The mechanisms of microorganism motility have been explored by the microbiology community^22^. High-resolution conventional microscopy was a key component in understanding of the mechanisms by which microorganisms move by aiding in the study of physiological and biological responses. In contrast, its use as an endogenous biomarker, especially in a high throughput context, has been understudied. There are significant advantages to utilizing the motility of different organisms for identification, especially in urine analysis. In the case of TV, motility exhibited through its flagella has been previously described as a corkscrew or zigzag motion^23^. Apart from microorganisms, there have also been extensive studies into the movement of RBCs in flow^24^. Due to the biconcave shape of the cells, they exhibit a flipping motion as they travel through a fluidic channel. This characteristic motion can be exploited for identification in low-resolution settings. There are inherent advantages of microfluidic lensless shadow imaging devices to study the use of motility as a contrast mechanism. For one, there is a large area over which the micro particles are allowed to move as shadow imaging has an inherently large depth of field and field of view. There is little risk of the organism travelling outside an observational area. It is a high-throughput system in which many particles can be tracked simultaneously; and the low resolution makes it necessary for motility to be a distinguishing feature. Microfluidic control allows for testing in pulsed flow to determine whether the particulates exhibit distinguishing features in still or moving flow. Finally, in contrast with holographic imaging, no image reconstruction in required.

This work presents the development of a low-cost lab-on-chip lensless optofluidic technology for the rapid point-of-care detection of urinary constituents. This technique utilizes the motion of urinary components as a biomarker and endogenous contrast mechanism, bypassing the need for the addition of molecular biomarkers or any sample preparation. In addition, such motion-based biomarker also circumvents the need for a high-resolution imaging modality, as the motion characteristics of the specimen can be analyzed easily in a low-resolution context. Certain components, like *Trichomonas vaginalis*, are self-propelled parasites that have their own inherent characteristic motility through the movement of their flagella, and others, like red blood cells, have their own distinct movement due to the flow in the channel. Shadow imaging provides a large field of view, which allows for the detection of rare events as the urine flows over the detector.

By filtering out negative samples early from the screening process, unnecessary culturing is avoided, as well as the potential pre-emptive prescribing of antibiotics. Ideally, this device would be implemented as a point-of-care device in clinics to reduce the number of samples being sent to the lab, as well as allowing for personalized medicine.

## Results

### Lensless optofluidic device

**Figure 1:**
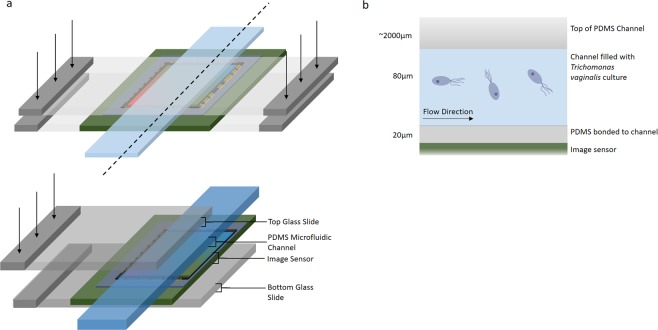
Overview of the Optofluidic Imaging Device. (**a**) A schematic of the lensless optofluidic device illustrates the different components and the implemented pressure mechanism. A PDMS microfluidic channel adhered to a plastic thin film sits atop an inexpensive and commercially available CMOS image sensor. A glass slide presses the channel to the sensor with enough force that the thin film beneath the channel is perfectly adhered to the sensor, ensuring no air bubbles are present. (**b)** A cross section of microfluidic channel illustrates the sample-sensor distance. The thin film adhered to the microfluidic channel is 20 μm in thickness and the height of the channel is 80 μm meaning the maximum sensor-sample distance is 100 μm. The channel is filled with culture and flown over the image sensor.

Typically, in an optofluidic projection imaging device, a microfluidic channel is bonded to an image sensor, and an incoherent light source, typically an LED, is placed above the senor. Samples are flown through the channel, in contact with the image sensor. In order to reduce the wear on the sensor and avoid cross contamination, this design features a removable flow channel module separate from the image sensor. The flow channel was clamped to the image sensor with a pressure-coupling mechanism, which also provided stability (Fig. 1). Microfluidic channels of varying heights were fabricated and tested, and a channel height of 80 μm was identified to be optimal. It allowed for the passing of all urinary constituents in the tested samples, without blockage. It also allowed sufficient resolution to identify the particulates within urine with the application of motion biomarkers. For the channel to be a self-contained replaceable module, it was bonded to a PDMS thin film 15 μm in thickness before being clamped to the sensor. The reduction in resolution due to the presence of the thin film beneath the microfluidic channel is not enough to render the algorithm unable to identify the particles. This channel is clamped to a low-cost, off-the-shelf, complementary metal-oxide-semiconductor (CMOS) image sensor with a 1.12 μm pixel size at a frame rate of 25fps over a field of view of 2.60 mm^2^. The channel fills the entire length of the sensor and 1 mm of its width, covering an active pixel area of 2.20 mm^2^. The illumination is provided by a broadband LED.

### Image and video processing

**Figure 2:**
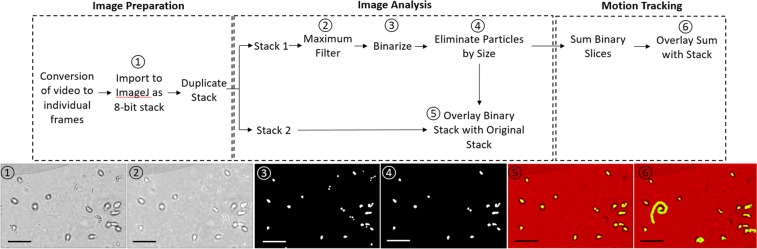
Tracking algorithm. The tracking algorithm follows all moving objects within the video, examples of which are highlighted in boxes. Scale 100 μm.

The tracking algorithm identifies each moving particle in the video, then tracks them as they move across the channel. The results are shown in Fig. 2 and in Supplementary Video 1. Once the particles are tracked throughout the video, the frames of the particulates can be extracted from within the bounding boxes to create a new image sequence to be analyzed for a motion biomarker. Our results show that such motion biomarkers can be used to classify red and white blood cells and *Trichomonas vaginalis*.

### Trichomonas vaginalis

**Figure 3:**
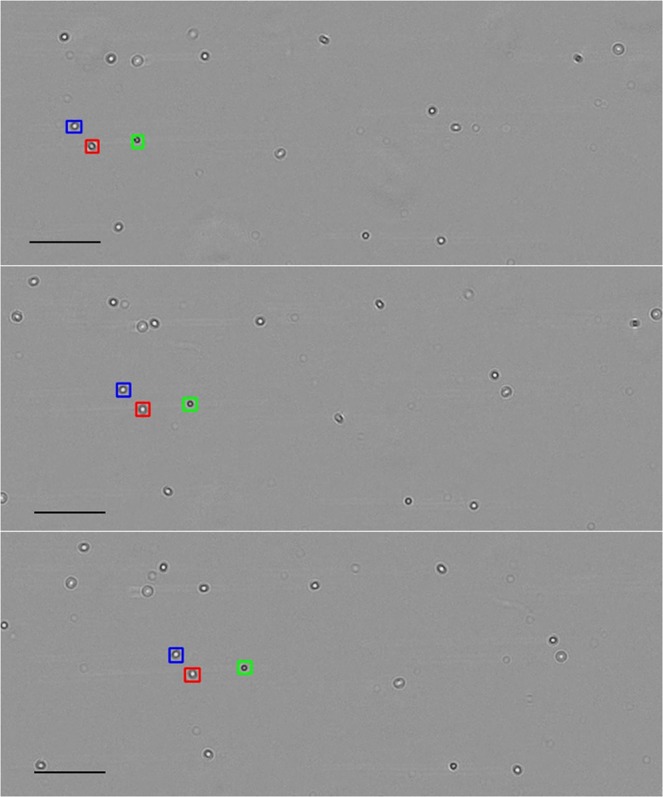
*Trichomonas vaginalis* identification and movement. (**a)** Original image of cultured *Trichomonas vaginalis* in the microfluidic channel at ¼ the field of view. TV appears elongated with a bright center and dark edges. Three individual TV parasites are boxed in the images. (**b)** Yellow indicates the identified *Trichomonas vaginalis* in the image post processing. Most TV parasites have been identified, however not in every frame due to the stretching and shrinking of the parasites over several seconds. (**c)** Indication of the path of the *Trichomonas vaginalis*. The parasites have a unique locomotion that can be seen when the frames are overlaid. This is a representation of the viability of the parasites in the sample. Scale 100 μm.

Positive control of cultured *Trichomonas vaginalis* (TV) was measured to validate the appearance of the parasite on the optofluidic microscope. The parasites are oblong in shape and can be up to 20 μm in length, which can be seen in Fig. 3a. On the lensless imaging platform, the parasites appear bright in the center due to the lensing effect of the parasites themselves, which focuses the incident light onto the detector. Similar effects have been seen in cyanobacteria as a mechanism to sense light direction^25^. When compared to other urine sediments, like bacteria and RBCs, TV is distinctly different due to its oblong shape and bright center. The closest particle in size is the WBC, which have nuclei that cast shadows at the center of the cell, and it is more spherical. The bright centers of the parasites as well as their large size and defined edges was the first defining feature used to identify them among the other particles present in the media. With this method, all particles that are the same size as TV with a bright center will be identified (Fig. 3b). While this is a sensitive method, it is not specific as TV detection is difficult in still images in which motility patterns are not visible^6^.

In clinical practice, morphology as well as the inherent locomotion of the parasites are used as identifiers in bright-field microscopy^6^. Supplementary Video 2 illustrates the movement of the TV in the channel when the flow is paused. Characteristic TV motility was confirmed independently by an experienced laboratory technologist. To recognize this type of motion, 200 frames of images were summed over 8 seconds of movement. Once summed, their movement pattern can be recognized. Some parasites moved in a zig-zag pattern, others in corkscrew patterns, as shown in the image (Fig. 3c). This is a distinctive identifier for TV. Other particles in a paused fluid would not have a motility pattern similar to that of this parasite.

Similar results were found when a urine sample was spiked with *Trichomonas vaginalis*. A true positive urine sample with *Trichomonas vaginalis* was unattainable as the parasites normally die within hours of sample collection.

### Blood cells

**Figure 4:**
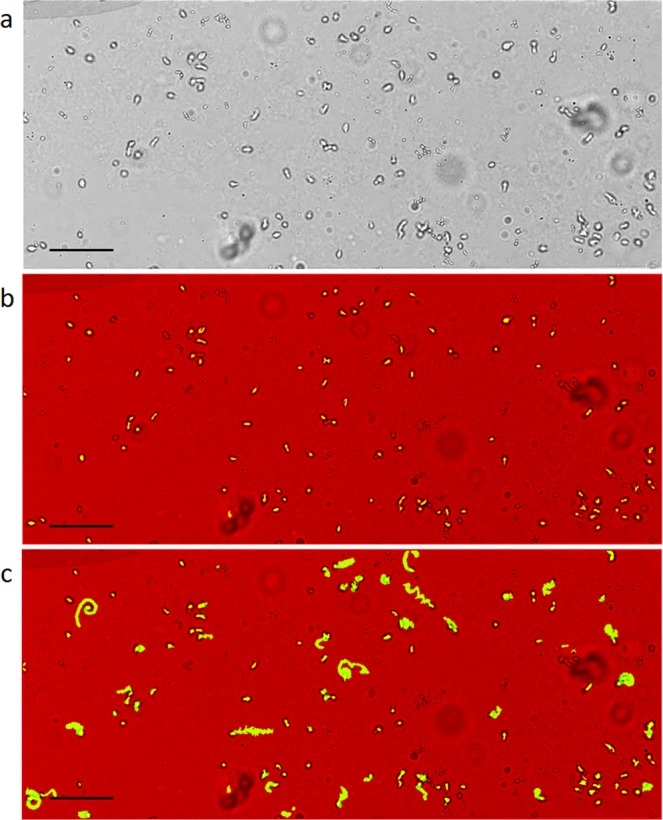
Images of diluted blood flowing through the channel. (**a)** Whole blood diluted in 1X PBS was flown through the microfluidic channel to investigate the motility and morphology of RBCs. A number of particles can be seen in the channel. Scale bar 80 μm. (**b)** The RBCs are biconcave in shape which causes them to flip as they flow through the channel. Although they have a characteristic morphology, it is not present in every frame. Scale bar 10 μm. (**c**) Other particles flowing through the channel do not exhibit the same flipping movement. Scale bar 10 μm.

In order to identify the different components in urine, homogenous samples were first tested. Human whole blood samples treated with anticoagulants were obtained from the microbiology Lab of HGS. The whole blood samples were diluted in 1× PBS pH 7 and flowed through the channel (Fig. 4; Supplementary Video 3). Some red blood cells (RBCs) are distinguishable through morphological features, particularly a divot in the center of the cell, which appears as a shadow. The biconcave shape of the RBCs and the laminar parabolic flow of the fluid through the channel causes the RBCs to flip repeatedly as they travel through the channel, as opposed to the rolling observed from other particles. Due to this motion, the RBCs do not appear to have this unique morphology in every frame (Fig. 4 inset). In some frames, the RBCs appear to be linear in shape as seen in Fig. 4b. In order to identify and accurately count the RBC’s in their flow, tracking and motion identification methods are employed.

**Figure 5:**
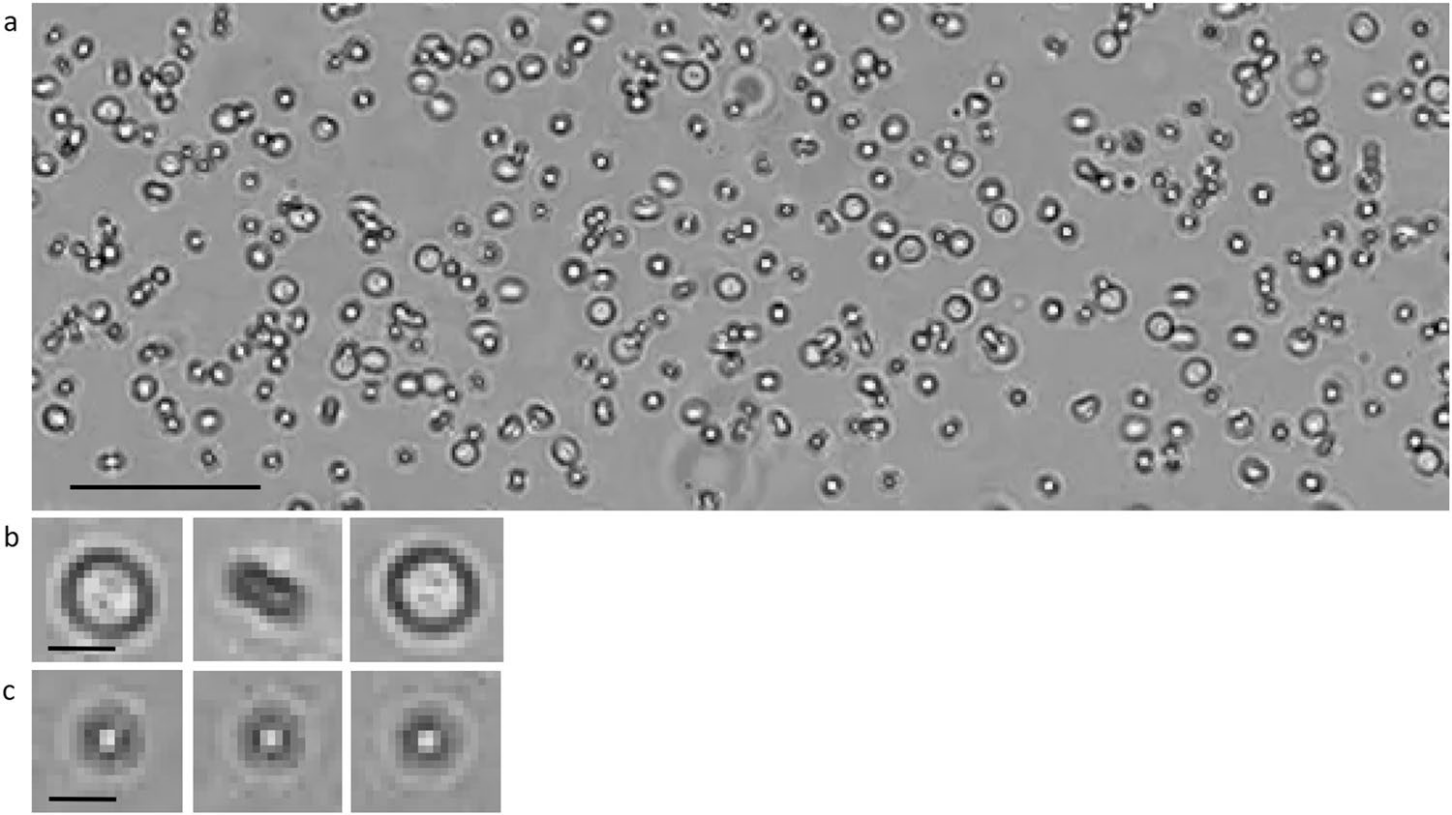
An RBC in a urine sample demonstrating flipping motion over five frames. The frames are made binary and an ellipse is fitted to the image of the RBC to estimate the elliptical ratio of the cell as it flips. This is then graphed against the frame number. A peak in the graph indicates a cell flipping. Scale 10 μm.

Urine samples from the HGH microbiology lab positive for hematuria were tested. Urine samples positive for RBCs were flown through the channel without preprocessing and RBCs were tracked and identified based on their rotational pattern. Background in these images was removed first, then an ellipse was fitted around the RBC. The major and minor axis of the ellipse were extrapolated from each cell, which is defined as the elliptical ratio of the RBC’s shadow image. The elliptical ration was then plotted as a function of frame number in Fig. 5 and is demonstrated in Supplementary Video 4.

**Figure 6:**
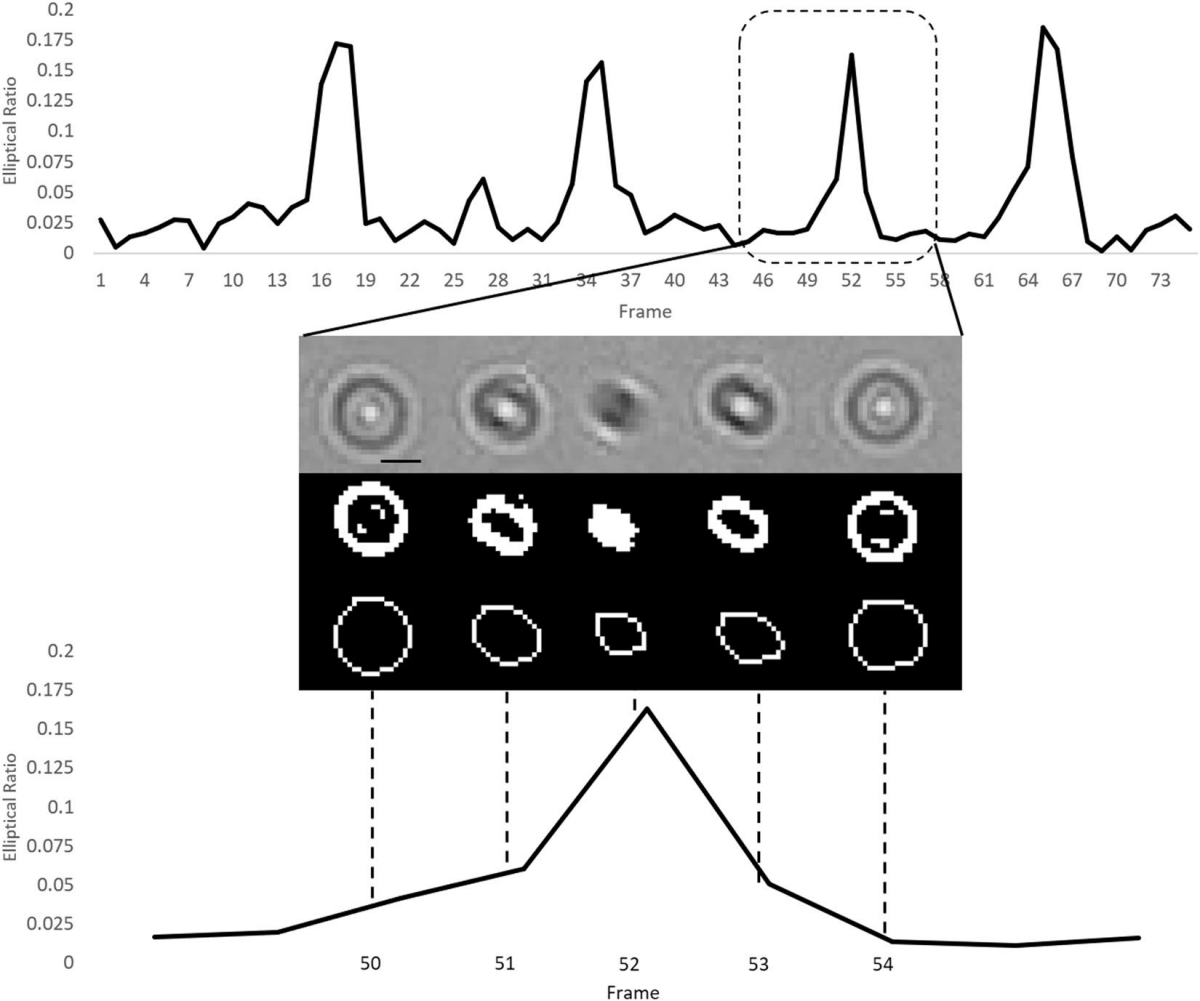
A white blood cell rolling through the channel. Due to the morphology of the white blood cells, they do not flip and thus do not exhibit the same pattern of movement when analyzed based on their major and minor axes. The resulting graph appears random, as there is no flipping of the cells through the channel. Peaks occasionally arise due to noise in the image that was not eliminated. The peak at frame 38 arose due to incorrect fitting of an ellipse over a WBC. Scale 10 μm.

The peaks indicate when the cell flips in the channel. When contrasted against white blood cells that roll through the channel, the same distinct pattern of peaks is not seen (Fig. 6). The WBCs were analyzed with the same algorithm as the RBCs. The only difference in the algorithm is that, with the WBCs, anything appearing in the stack that was smaller than 50 pixels was eliminated. This is due to the size difference between the WBCs and RBCs.

Although RBCs are normally reported to be around 6–8 μm in diameter, the RBCs in an 80 μm channel appear to be around 15 μm. The shadows are enlarged due to the height of the channel and the position of the objects within the channel. The further away the sample is from the sensor, the larger and less clear the sample appears. The WBCs are also enlarged, as they appear to be around 30 μm as opposed to the reported 12–17 μm. This discrepancy between the real size and morphology of the cells versus how they appear on the detector indicate that it is not a reliable method of identification. By analyzing and applying the flipping motion of the cells, a more differentiable characterization can be executed.

In addition, the flow of the particles through the channel is not uniform. Due to the laminar parabolic flow in the channel, the particles exhibit a different flow speed based on their position in the channel. The image size and the flow speed of objects in the images can be used to calculate the actual size and height of the particle^26^. The number of blood cells in urine can be counted without sample preparation, however as there is a much higher concentration of blood cells in blood and the blood sample must be diluted if this platform should be extended to hemocytometry. Upon testing urine samples, other components were also found with examples shown in Supplementary Fig. 1.

## Discussion and Conclusion

In this work, we developed a new, low-cost, reusable, lensless imaging platform for the clinical analysis of urine samples. Shadow imaging, in combination with motion analysis as an endogenous biomarker, leads to a unique application. To our knowledge, this is the first report of lensless imaging for urine analysis as well as the first application of cell and organism motility as a biomarker in lensless shadow imaging. This device demonstrates effective detection of blood cells and parasites directly in urine samples without the need for concentration or culture. TV self-propel through the movement of their flagella, often resulting in a corkscrew or zig-zag movement. Red blood cells have a distinct flipping movement due to the flow in the channel and their biconcave morphology. In this context, shadow imaging is in a unique position to take advantage of this unique motility for particle identification.

An important advantage of shadow imaging is that the images acquired do not require extensive post processing or reconstruction. It is normally well suited for the imaging of biological specimen, in which the samples have some degree of transparency. Recently, holographic imaging is a very popular lensless imaging technique where a diffraction image is projected onto the sensor. Although holographic imaging has the advantage of reconstructing different planes in a 3D volume, it has challenges in real time imaging of a deep (~50–100 μm) microfluidic flow channel due to the lengthy processing time. We demonstrated that shadow imaging has the specific advantage of being able to be used in combination with motility biomarkers to specifically identify urine sediments.

In terms of the lensless imaging device design, the use of a clamping system allows for the easy replacement of microfluidic channels between samples without having to replace the image sensor. Disposable sample holders are important in clinical use to mitigate cross-contamination. Typically, a polydimethyl-siloxane (PDMS) microfluidic channel is adhered to the sensor through plasma bonding to ensure a minimal sample-sensor distance and high resolution. This method reduces the reusability of the device, as both the channel and the imager need to be replaced after each test, significantly increasing the cost. In addition, in typical shadow imaging devices, the height of the device is often constrained to the size of the particles to ensure that they remain close to the sensor as they flow through the channel. Due to the nature of urine, a fluid sample with a large diversity of constituents in both size and shape, a channel must be fabricated such that the largest of particles can pass through. Although the resolution of particles is best when the sample-sensor distance is highly reduced, our study illustrates that a larger distance of 20 μm–100 μm does not negatively affect particle identification. The image sensor, with a pixel size 1.12 μm, allows for a relatively high resolution in the context of shadow-imaging devices. The use of a microfluidic channel allows us to continuously screen for pathogens in the sample. At its height of 80 μm, it is able to hold 0.172 μl over the field of view of ~2.15 mm^2^. This height allows all of the components of urine and blood to pass through without issue and retains the resolution necessary to identify the pathogens. Reconstruction of the images is not necessary, and a sufficient resolution is achieved for the identification of the components. Furthermore, the use of a Raspberry Pi microcontroller and associated camera significantly simplified the integrated device and greatly reduced the total system cost to $50, suitable for applications in low resource areas.

In the current setup, the focus is to study the detection mechanism. Therefore, the samples were pushed through the syringe manually. Due to the manual injection of the samples in the microfluidic channel, there are some irregularities in the flow over time. For final point-of-care applications, a pumped flow system should be implemented independent of the user. A syringe pump (NE-1002X, New Era Pump Systems, Farmingdale, NY) was used in attempt to keep the flow through the microfluidic channel constant, however it was unable to do so. Low flow rates, below 50 ul/min did not push fluid through the channel, and higher flow rates, greater than 50ul/min, pushed the sample through too quickly. We plan to explore other sample loading processes in order to ensure test stability in future work. Five channel designs with heights of 20 μm, 50 μm, and 80 μm were tested for this application. The channel that was chosen for the final design was a rectangular channel with an 80 μm height and a width of 1 mm in order to cover the active pixels on the sensor. The resulting flow rate used in the experiments described in this manuscript was calculated to be ~3 μl/min. The length of the channel was enough to cover the sensor and extend slightly past it on both sides to accommodate the inlet and outlet holes outside of the field of view. The main goal of this project was imaging capability and a high throughput, thus a more complicated channel was not investigated. This design choice allowed for unperturbed flow (no clogging from the larger urine sediments) and covered the greatest active pixel area. The channels with a 20 μm height collapsed and the top of the channel adhered to the thin-film PDMS, blocking all flow. The channels with a 50 μm height required a great amount of pressure to flow fluids through and did not result in a higher resolution in comparison to the 80 μm channels. In the 80 μm channel, the particles are able to travel along the top of the channel (far from the sensor) or at the bottom of the channel (close to the sensor). When closer to the sensor, smaller features are distinguishable on the particles. As a result, the spatial resolution of the device varies depending on the height position of the target. In this work, we explored a number of targets with different sizes and morphologies. The smallest specimen tested in the channel was Saccharomyces cerevisiae with a diameter ranging from 3–4 μm. The cell membrane was clearly defined in the images. Bacteria and 1 μm beads were also tested on the system, with the channel and with a plastic thin film (data not shown). In the channel, both the beads and the bacteria were too small to identify with certainty. On the plastic thin film, there was a resolution increase due to the proximity to the sensor, however individual bacterium remains difficult to be visualized directly. Pixel super-resolution methods have been explored by a number of groups^19,27,28^. Motion based image analysis methods are being explored in an ongoing project with the hope to provide identification beyond the direct resolution limit.

We developed a tracking algorithm to identify large (>2 μm) objects in the flow channel. Once each object is identified, a video of its movement with the flow is used in the motion analysis. Currently, the algorithm misses approximately 15% of objects in comparison with visual identification. Such an issue may be caused by (i) edge detection missing particles or finding false particles, and (ii) uncertainties in the tracking due to two adjacent particles. A 3D Convolutional Neural Network could be used to reduce false results obtained from edge detection^29^. To improve the accuracy of tracking, known particle features obtained from previous frames can be involved in an improved tracking algorithm, such as movement direction and speed. This tracking algorithm can be used in sequence with a classification algorithm arising from the motion biomarkers of each component.

To classify each particle, distinguishing features must be used. Typically, the morphology of different cells is an identifying feature that can be used to distinguish one from another. However, on an imaging platform with a lower resolution, the morphology alone may not be enough to distinguish different particulates^30^. Motility of microorganisms on a lensless imaging device is not a novel concept. Zhang *et al*.^31^ investigated the motility of sperm in two dimensions through lensless shadow imaging in a microfluidic channel for viability testing. Di Caprio *et al*.^32^ continued the investigation of sperm motility through lensless holographic shadow imaging in four dimensions. Both of these significant studies investigated cell tracking with lensless imaging, however, unlike in our study, this was done to study the viability of the cells, not identify them in a mosaic of other components. RBCs in whole blood and found natively in urine illustrates the flipping motion characterized extensively in prior work^24^. By analyzing the flipping of the cells in the channel, an algorithm can be developed for automatic detection. The elliptical ratio of the RBCs, as they flip through the channel, is distinct from that of the WBCs, indicating a unique biomarker. These results demonstrated, in principle, RBCs can be identified without staining through the use of motility and tracking. Huang *et al*.^28^ developed a lensless hemocytometer that relies on single frame pixel super resolution techniques to enhance the resolution of the lensless images in order to identify the cells present through their morphology. Our method presents an alternative to this machine learning technique, which may result in less computational power. We also demonstrate that an increase in resolution is not necessary for the identification of the blood cells. Lee *et al*.^33^ developed a hemocytometer for the analysis of blood cells in cerebrospinal fluid. They use a high microfluidic channel (532 μm) and require a 10 minute sedimentation period in which the flow is paused to allow the cells to drift to the bottom on the channel, toward the sensor, to attain a higher resolution for morphological identification. They are also able to stain their samples for fluorescence imaging. In our design, there is continuous flow in the channel to allow for a high throughput, and there is no need for resolution enhancement.

We have demonstrated that *Trichomonas vaginalis* can be identified based on its size and bright center. Motion analysis arises through frame accumulation, in which the unique corkscrew motion, a measure of viability can be seen, which is similar to what others have reported for other motile parasites^34^. Increasing the amount of urine being screened on the device increases the limit of detection. Trichomoniasis is typically diagnosed through wet mount microscopy where anywhere between <1 and 16 parasites can be found per high power field of 60x^4^. The field of view of a 60x image can be approximated to be 0.03 mm^2^, which is far less than the field of view of the presented microscope (2.6mm^2^). It is likely that very low amounts of Trichomonas vaginalis can be detected with this platform as there is continuous flow of the samples and the parasite is fairly large and distinguishable. In addition, due to the low-cost of the system, it is implementable at the site of sample collection. This gives us the opportunity to analyze the motion of the parasite when it is at its liveliest.

Crystals and casts are very large and easily identifiable based on their morphology. These characteristics can then be used to train an algorithm to automatically identify the specimen. The next step in the development of an accurate diagnosis tool is to be able to use the identifiable characteristics of each component to rapidly and accurately analyze each particle.

Successful development and implementation of this lensless imaging platform has the potential to keep the cost of diagnosis low and allow for timely feedback. We have demonstrated that lensless optofluidic projection imaging is able to simultaneously detect various pathogens in urine. The implementation of a fully automated lensless imaging platform can quickly eliminate negative samples from further processing to significantly reduce costs; and administer earlier and more appropriate treatments. Such features fit the application of point-of-care diagnosis in hospitals, clinics and long term care facilities. This work provided the initial steps into the development of this platform.

## Materials and Methods

In this platform, a fluid sample is flowed within a microfluidic channel directly over a CMOS image sensor, which captures a series of projection images. It is then processed with an automated detection algorithm.

### Device design and fabrication

As shown in Fig. 1, the lensless optofluidic shadow imaging device consists of a polydimethyl-siloxane (PDMS) microfluidic channel of 1 mm width and 80 μm height, with an inlet and outlet hole, bonded to a spin coated thin film PDMS, 15 μm in thickness. This channel is clamped to a low cost off the shelf complementary metal-oxide-semiconductor (CMOS) image sensor (IMX219PQ, ¼”, 3280 × 2464 8.08 M pixels, back-illumination, Sony), with a 1.12 μm pixel size. The image sensor is commercially sold as a part of the Pi v2 camera and is controlled by a Raspberry Pi 3 single board computer. This clamping system allows the image sensor to be reusable as the channel can easily be switched out. Pieces of electrical tape are placed underneath the inlet and outlet holes to prevent the tubing or any pressure from breaking the film. The channel height allows for all components in the urine sample to flow through without causing blockage. The light source illuminating the platform originates from an incoherent 1 W white LED placed 30 cm above the sample. The lamp (003.859.41, Ikea) faces vertically downwards, directly over the image sensor and the diameter of the area of illumination is approximately 30 cm resulting in average intensity of 1.4 mW/cm^2^. For imaging, a liquid sample is dispensed from a syringe and into the microfluidic channel. Samples were typically imaged at a frame rate of 25 fps. The camera can be operated at a slower frame rate of 15 fps in order to achieve a resolution of 2592 × 1944 pixels, or at a faster frame rate of up to 90 fps with a field of view (FoV) of 940 × 480 pixels. The FoV of the sensor at 25fps is 2.60 mm^2^. The flow channel, which is 1 mm in diameter, covers an area of 2.15 mm^2^. At a channel height of 80 μm, it is able to hold 0.172 μl over the field of view. The optofluidic microscope integrates microscale fluidics and optics in a single system to detect the different components of urine without pre-processing of the sample.

### Biological sample preparation and measurements

In order to identify the different components in urine, three samples were tested: whole blood, Trichomonas vaginalis (TV), and patient urine samples. These samples were received from the Clinical Pathology Lab of HGS.

#### Clinical blood specimens

Whole blood samples were obtained from healthy patients from remaining transfusion medicine. Samples were stored at 4 °C until testing. Samples were diluted by a factor of 1:100 in 1x phosphate-buffered saline (PBS) pH 7 for testing.

#### Urine samples

All urine samples were acquired and handled according to the protocols approved by the Hamilton Integrated Ethics Board (HiREB). Urine samples were received and processed at Hamilton General’s Clinical Pathology lab through culture on the Walk Away Specimen Processor (WASP). After evaluation through culture, throughout which the urine samples were stored at 4 °C for approximately 3 days, the samples were then tested on the lensless imaging platform.

#### Trichomonas vaginalis

TV was cultured from a patient in modified Diamonds medium and incubated at 37 °C.

### Sample loading process

The samples were then manually injected into the microfluidic channel under white-light illumination. Images of the blood cells are captured by the image sensor. The whole blood and urine samples were manually injected with a syringe and travelled at a rate of 500 μm/sec. TV was injected into the channel and allowed to rest without flow in order to analyze the locomotion of individual parasites.

### Image processing

In order to identify the urinary constituents in the acquired image sequences, a tracking algorithm was developed to highlight each moving particle in the video. Once each moving object is identified, they are tracked as they move across the channel. The frames of the particulates are then extracted to create a new image sequence to be analyzed for a motion biomarker. Once the particulate matches a motion biomarker, it can be properly classified.

#### Tracking

The tracking algorithm was developed in Python with the OpenCV image processing package to highlight each moving particle in the video. The developed algorithm applies the Gaussian mixture-based background/foreground segmentation algorithm and morphological transformations to remove background as well as non-moving objects in the video^35^. Edge detection is then used to detect moving objects in each frame. Once a particle is detected, both edge detection and the discriminative correlation filter are used to track it over consecutive frames. The Kalman filter is used to predict the position of particles if overlapping were to occur.

#### Trichomonas vaginalis

**Figure 7:**
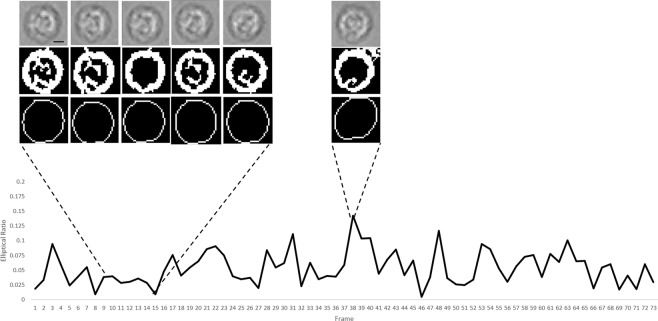
Image processing sequence for *Trichomonas vaginalis*. This sequence identifies the TV in the channel and highlights its movement across frames. Scale bar 50 μm.

Figure 7 outlines the image processing flow for TV in Fiji/ImageJ (v1.52i)^36,37^. After using FFmpeg^38^ to convert the video to individual frames, a maximum filter was used to enhance the brightness of the center of the parasites. The images were then binarized and white areas smaller than 20 pixels were eliminated as this is not representative of the size of TV. This new stack can be overlaid with the original image stack to identify the parasites. It can also be summed together for motion analysis.

#### Blood cells

In order to determine whether this signature exists in RBCs present in urine, urine samples from the HGH microbiology lab positive for hematuria were tested. Urine samples positive for RBCs were flown through the channel without preprocessing and RBCs were tracked and identified based on their rotational pattern. A stack of images following an RBC travelling through the channel was analyzed using ImageJ. The stack was averaged, and the averaged frame was subtracted from the stack to remove background noise. The stack was then converted to binary. Using the built-in Analyze Particles tool in ImageJ^36,37^, anything appearing in the frame stack that was smaller than 20 pixels was eliminated. The major and minor axis of the cell as it travelled through the channel was extracted and the elliptical ratio was defined using Eq. 1. The same process was done for WBCs.

The major and minor axis of the ellipse were extrapolated from each cell. It is important to note that the eccentricity of the cells themselves remain the same, but the shadow projected onto the detector can be analyzed by tracking and comparing the changes between the major and minor axis. The elliptical ratio of the RBC’s shadow image is defined in Eq. 1 to capture the difference of major axis to minor axis in the shadow image:

1 $$Elliptical\,Ratio=\frac{Major\,Axis-Minor\,Axis}{Major\,Axis+Minor\,Axis}$$

### Research ethics

A human tissue sample research ethics protocol including informed consent has been developed and approved by the Hamilton Integrated Ethics Board (HiREB approval #2062) covering both McMaster University and its affiliated Hospitals (Hamilton General Hospital/HGH, Hamilton Health Sciences/HHS). All methods were performed in accordance with the relevant guidelines and regulations. Informed consent was obtained when required.

## Supplementary information

supplementary list

Supplementary Video 1

Supplementary Video 2

Supplementary Video 3

Supplementary Video 4
