# Author Correction: The Use of Motion Analysis as Particle Biomarkers in Lensless Optofluidic Projection Imaging for Point of Care Urine Analysis

**DOI:** 10.1038/s41598-020-65965-3

**Published:** 2020-06-04

**Authors:** Jessica Kun, Marek Smieja, Bo Xiong, Leyla Soleymani, Qiyin Fang

**Affiliations:** 1 School of Biomedical Engineering, McMaster University, Hamilton, ON Canada; 2 Department of Pathology and Laboratory Medicine, McMaster University, Hamilton, ON Canada; 3 Department of Engineering Physics, McMaster University, Hamilton, ON Canada

Correction to: *Scientific Reports* 10.1038/s41598-019-53477-8, published online 21 November 2019

This Article contains an error in the order of the Figures. Figures 2, 3, 4, 5, 6 and 7 incorrectly appear as Figures 3, 4, 5, 6, 7 and 2, respectively. The order of Figure legends is correct.

Figures 1–7 appear below in their correct order.

**Figure 1:**
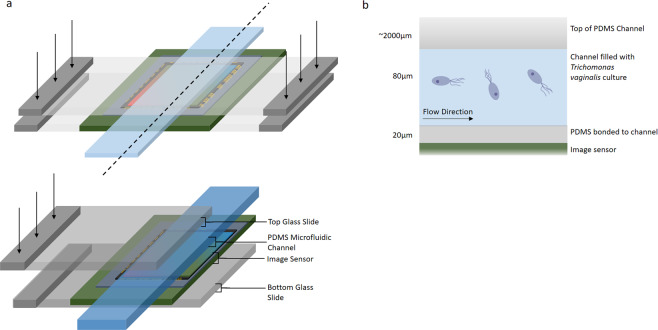
.

**Figure 2:**
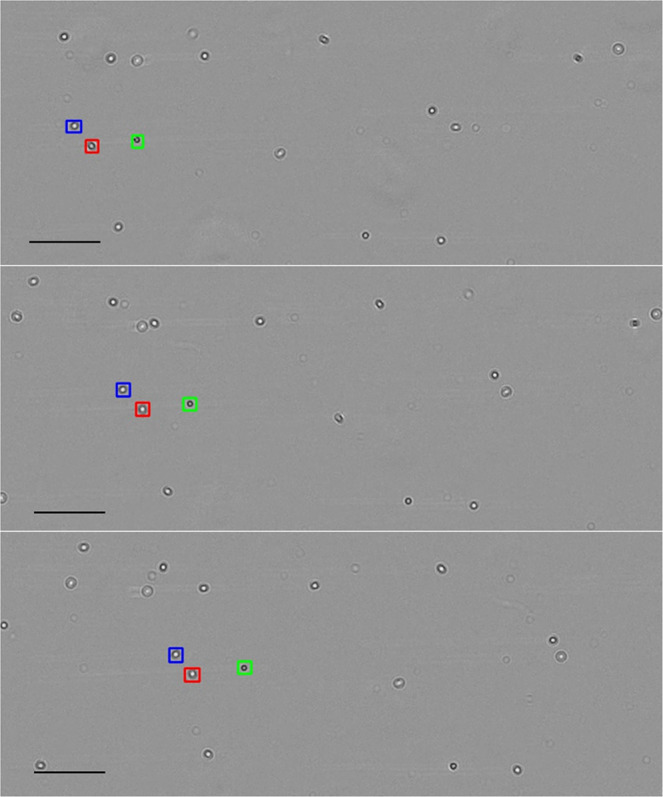
.

**Figure 3:**
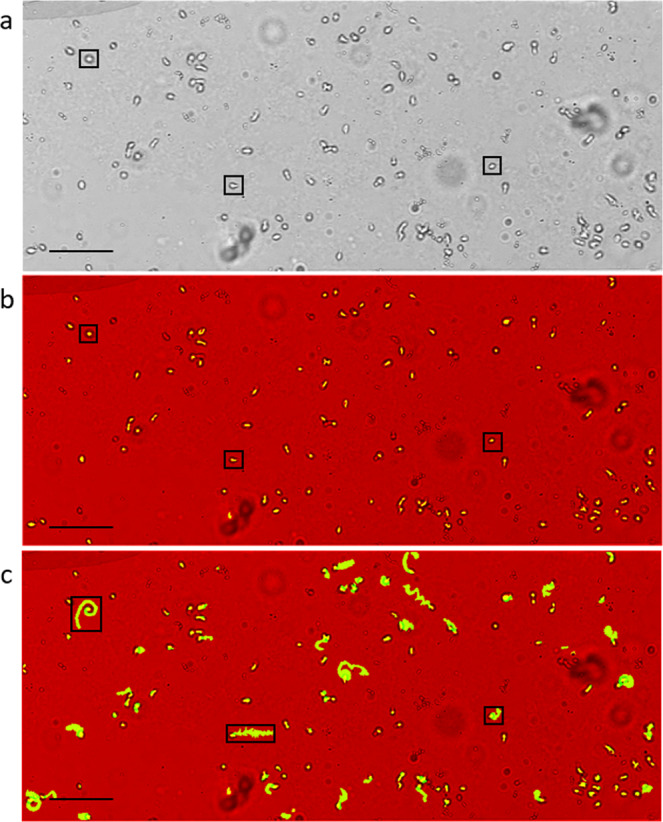
.

**Figure 4:**
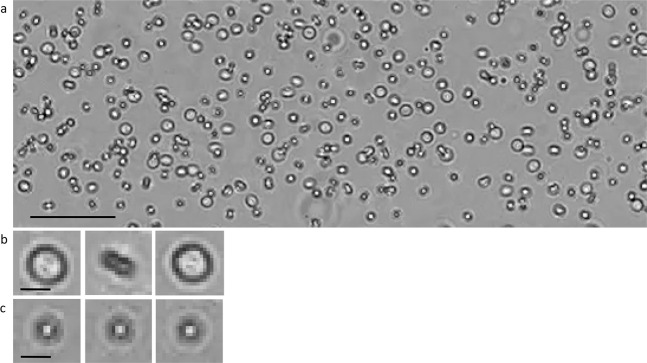
.

**Figure 5:**
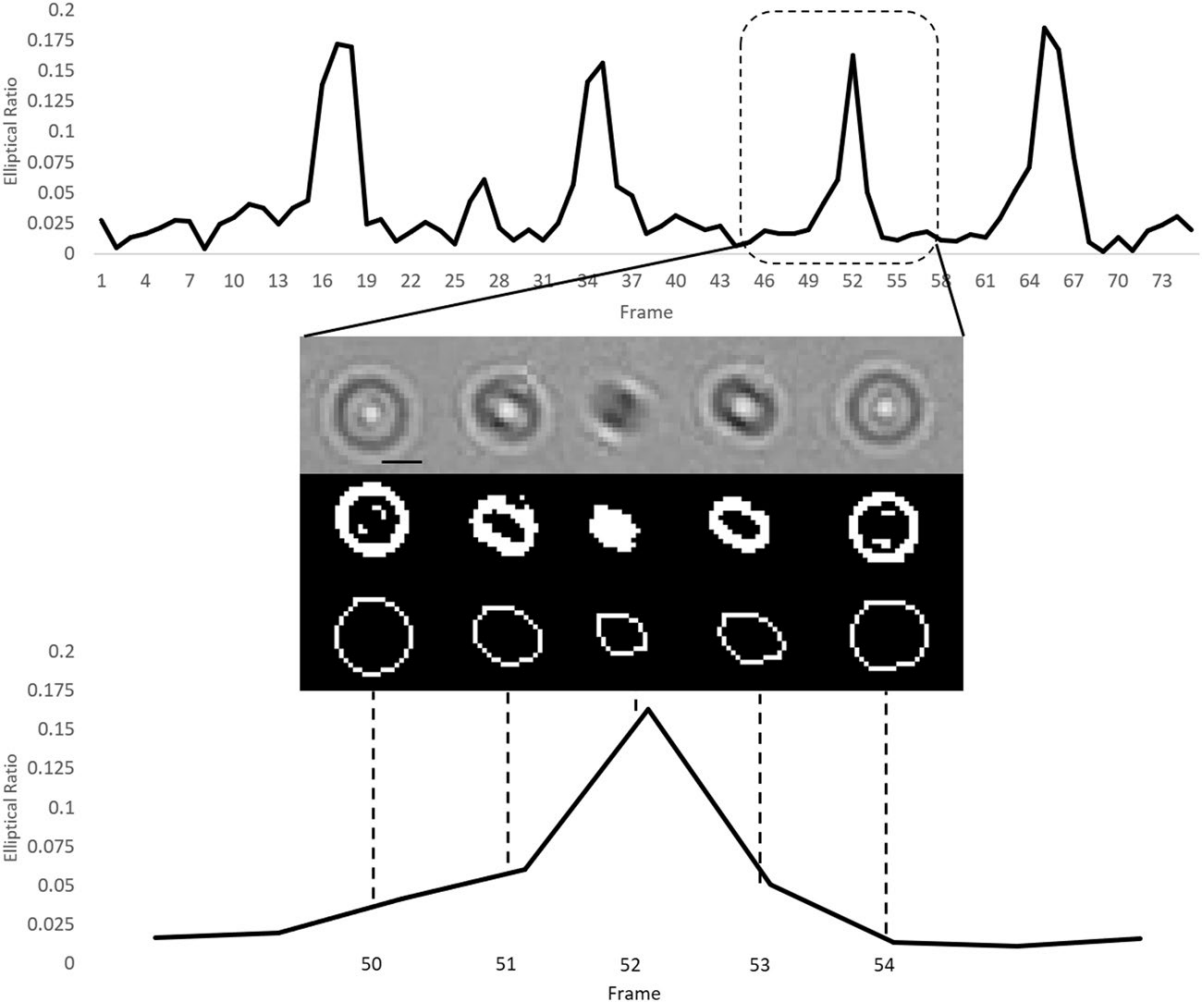
.

**Figure 6:**
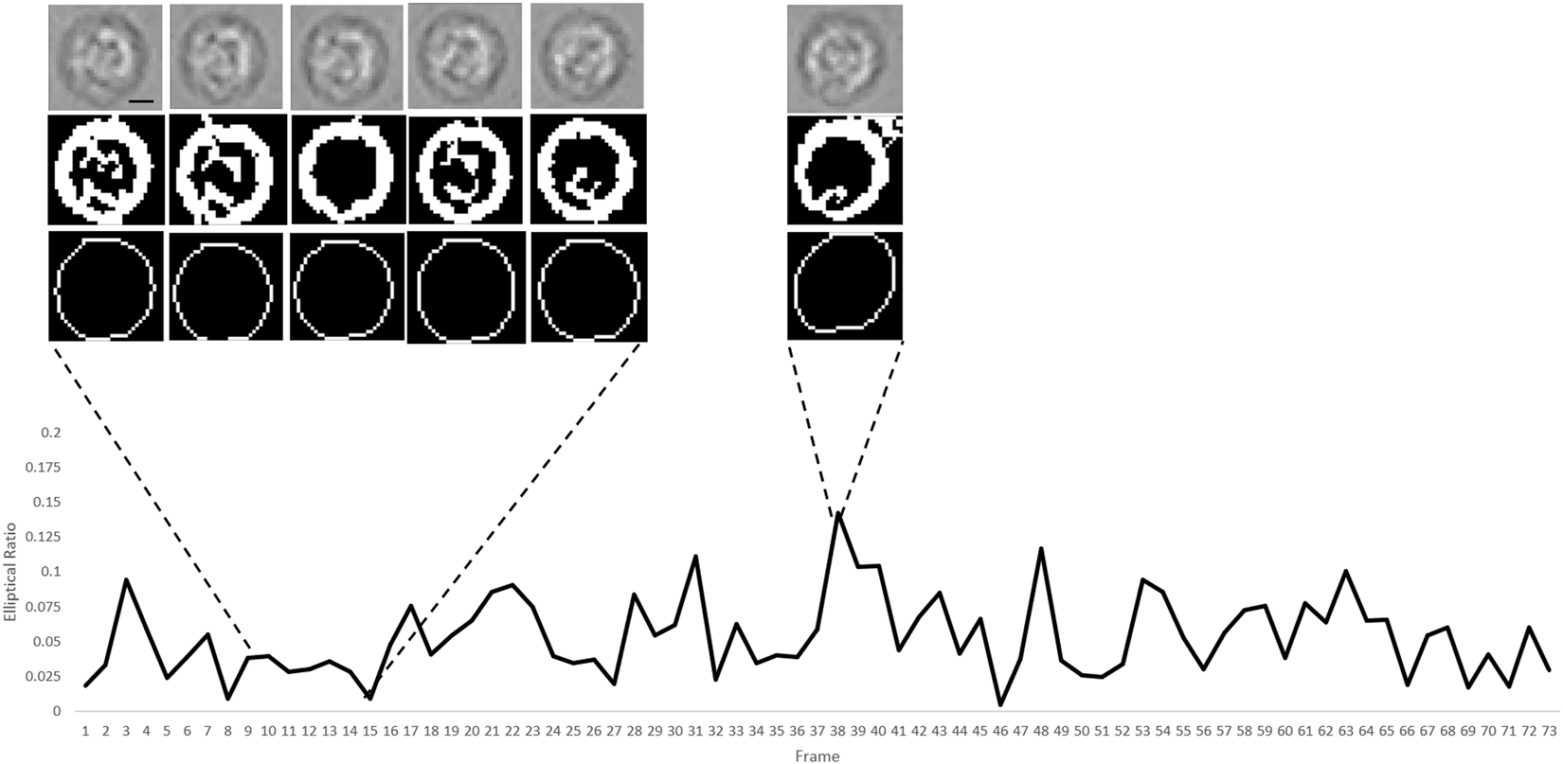
.

**Figure 7:**
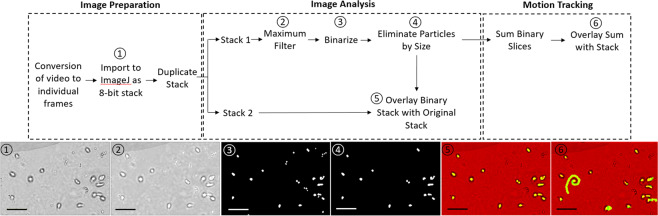
.

