# Supplementary material for: The Use of Motion Analysis as Particle Biomarkers in Lensless Optofluidic Projection Imaging for Point of Care Urine Analysis
Source: Sci Rep. 2019 Nov 21;9:17255. doi: 10.1038/s41598-019-53477-8 (PMC6872526; doi:10.1038/s41598-019-53477-8)

The Use of Motion Analysis as Particle Biomarkers in Lensless Optofluidic Projection Imaging for Point of Care Urine Analysis

Jessica Kun[1], Marek Smieja[2], Bo Xiong[1], Leyla Soleymani[1,3], Qiyin Fang[1, 3*]

[1] School of Biomedical Engineering, McMaster University, Hamilton, Ontario, Canada

[2] Department of Pathology and Laboratory Medicine, McMaster University

[3] Department of Engineering Physics, McMaster University

**Corresponding author: qiyin.fang@mcmaster.ca*

Supplementary Video and Figure Captions

Supplementary Figure 1| Projection Images of Sediments Found in Urine. a. Haline casts have been identified in a patient's urine through their size and morphology. This is comparable to brightfield microscopic urinalysis and different casts are likely able to be distinguishable. **b.** Squamous Epithelial Cells have also been identified based on morphology **c.** Baker's Yeast, or *Saccharomyces cerevisiae* tested after being dissolved in water. The resolution of the shadow image is sufficient to recognize their oblong shape **d.** Crystals are also evident in patients' urine through morphological features.

Supplementary Video 1| Tracking algorithm. The first frame with the scale bar is found in Fig. 2 of the main article. The tracking algorithm follows all moving objects within the video, examples of which are highlighted in boxes. Large distortions in the video are artifacts from background noise removal but are not tracked by the algorithm.

Supplementary Video 2| ***Trichomonas vaginalis*** **identification and movement.** The first frame with the scale bar is found in Fig. 3 of the main article. Original video of cultured *Trichomonas vaginalis* in the microfluidic channel at ¼ the field of view. TV appears elongated with a bright center and dark edges and tends to undergo a slow spinning motion. They are often identified through this inherent corkscrew locomotion.

Supplementary Video 3| Images of diluted blood flowing through the channel. The first frame with the scale bar is found in Fig. 4 of the main article. Whole blood diluted in 1X PBS was flown through the microfluidic channel to investigate the motility and morphology of RBCs. A number of particles can be seen in the channel. Scale bar 80µm. The RBCs are biconcave in shape which causes them to flip as they flow through the channel. Although they have a characteristic morphology, it is not present in every frame.

Supplementary Video 4| An RBC in a urine sample demonstrating flipping motion over five frames. The first frame with the scale bar is found in Fig. 5 of the main article. The frames are made binary and an ellipse is fitted to the image of the RBC to estimate the elliptical ratio of the cell as it flips. This is then graphed against the frame number. A peak in the graph indicates a cell flipping. The RBC is aligned with the graph as it flips through the channel.

Supplement Figure 1

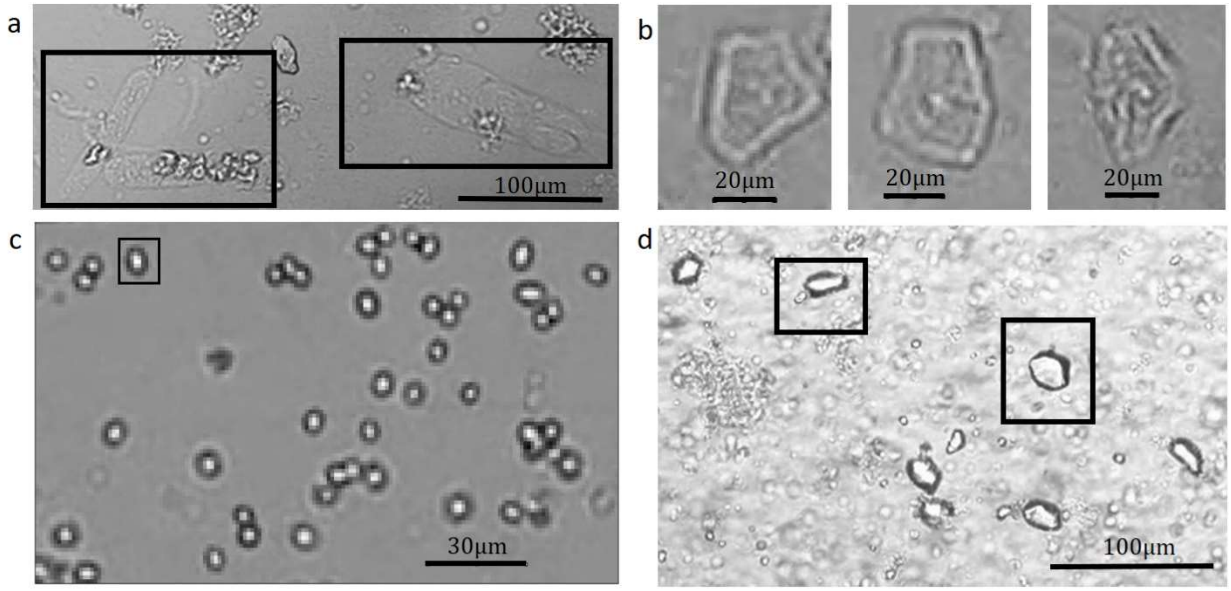

Supplement: Supplementary file 1 — supplementary list [file 41598_2019_53477_MOESM1_ESM.pdf]
